# Effect of metal artifact removal modes on the accuracy of linear measurement around titanium implants by applying different voltages: an original article

**DOI:** 10.1186/s12880-023-01025-2

**Published:** 2023-06-05

**Authors:** Daryoush Goodarzi Pour, Elham Emami Meybodi, Kosar Sodagar, Zahra Alsadat Emami Meybodi, Sanaz Safari

**Affiliations:** 1 Department of Oral and Maxillofacial Radiology, School of Dentistry, Tehran University of Medical Sciences, Tehran, Iran; 2 School of Dentistry, Tehran University of Medical Sciences, Tehran, Iran; 3 School of Dentistry, Qazvin University of Medical Sciences, Qazvin, Iran

**Keywords:** Artifacts, Cone-beam computed tomography, Implants, Titanium

## Abstract

**Background:**

This study aims to evaluate the effects of the artifact removal algorithm on linear measurements of the buccal cortical plate by altering the voltage.

**Methods:**

Ten titanium fixtures were inserted at the site of central, lateral, canine, premolars and molars of dry human mandibles. Vertical height of buccal plate was measured using a digital caliper as a gold standard. Mandibles were scanned with 54 and 58 kVp. Other parameters were constant. Images were reconstructed with none, low, medium and high artifact removal modes. Two Oromaxillofacial radiologists evaluated and measured the buccal plate height using Romexis software. Statistical package for the social sciences (SPSS) version 24 was used for data analysis.

**Results:**

In medium and high modes, the difference between 54 and 58 kVp was significant (*p* < 0.001). No significance was noted by using low ARM (artifact removal mode) at the 54 kVp and 58 kVp.

**Conclusion:**

Using artifact removal in low voltage decreases the accuracy of linear measurement and buccal crest visibility. By using high voltage, artifact removal would have no significant effect on accuracy of linear measurements.

## Background

Cone-beam computed tomography (CBCT) imaging is recommended for implant placement [1–3]. This modality enables three dimensional (3D) assessment of anatomical structures with an exposure dose less than that of other 3D imaging modalities such as computed tomography (CT) [2, 4, 5]. Nowadays, CBCT imaging modality is utilized more than before to assess peri-implant hard tissue, especially in the aesthetic zone, with the risk of resorption of a thin buccal plate [2, 6, 7]. However, quality reduction due to metal artifacts around high-density objects significantly decreases the accuracy of this technique [8–10]. This type of artifact is referred to as the beam hardening artifact and appears as dark bands and streaks and cupping artifact around metal objects [10–12]. In fact, these objects absorb a high portion of X-ray photons due to high density and create artifacts [5, 10, 11]. On the other hand, the accuracy of linear measurements and visibility of anatomical structures such as the alveolar crest are highly important since they can significantly affect the treatment plan [13–15].

There are different ways to minimize artifacts such as using anti-scatter guard, small field of view (FOV) and changing the exposure setting [5, 10, 16–18]. Moreover, the manufacturers have introduced algorithms such as artifact removal modes (ARMs) to decrease the metal artifacts [2, 8, 10, 11, 19].

Although some studies have evaluated the efficacy of such algorithms, no previous study has assessed the effect of metal artifact removal (MAR) algorithms on the accuracy of linear measurements made on CBCT scans taken at different voltage values, which is the aim of this study [2, 8, 10, 16, 20, 21]. There is a possibility that these algorithms eliminate beneficial data [8].

## Methods

**Fig. 1:**
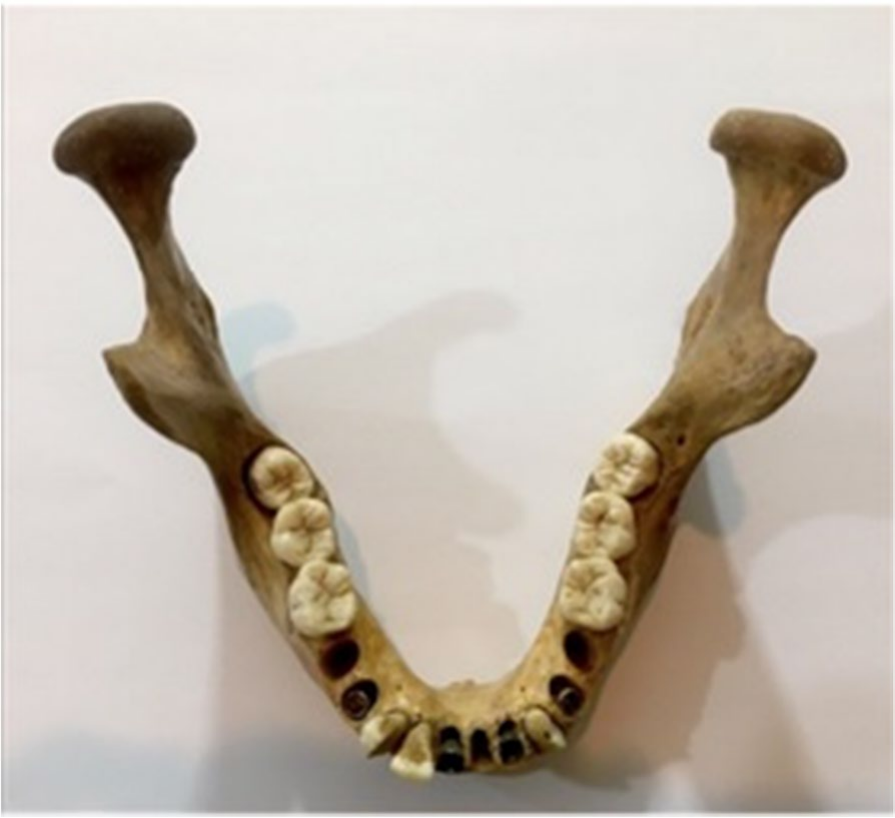
Dry mandible containing dental implants

In this experimental study, 10 titanium fixtures 4 × 8 mm (Super Line; Dentium, Implantium, Seoul, Korea) were inserted in two dry human mandibles obtained from the Anatomy Department of Tehran University of Medical Sciences which are used for scientific research and no patient have been involved in this study. The approval ID of Ethics committee is IR.TUMS.DENTISTRY.REC.1396.2970. An expert oral and maxillofacial surgeon placed dental implants at the alveolar crest level in the central and lateral incisors, canine, premolars, and molars (Fig. 1).

**Fig. 2:**
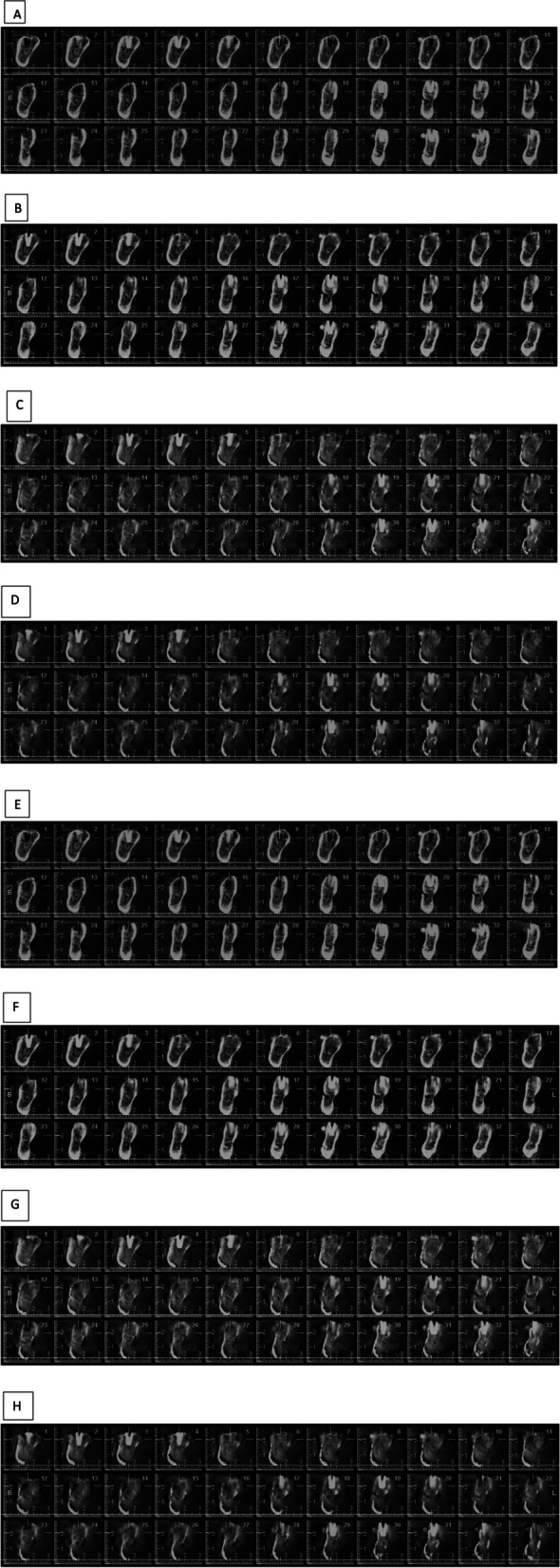
Sample of reconstructed images using different artifact removal modes with two voltages: **A** None ARM, 58 KVP, **B** Low ARM, 58 KVP, **C** Medium ARM, 58 KVP, **D** High ARM, 58 KVP, **E** None ARM, 54 KVP, **F** Low ARM, 54 KVP, **G** Medium ARM, 54 KVP, **H** High ARM, 54 KVP

Both mandibles were fixed on an acrylic platform and then scanned by ProMax CBCT system (Planmeca, Helsinki, Finland) with a FOV of 8 × 8 cm. The exposure parameters were the same as 5 mA and 12.42 s at 54 and 58 kVp voltage values. Arc rotation was 270° and voxel size was 0.32 mm. There were four modes of artifact removal algorithms employed in the image reconstruction (Fig. 2): no artifact removal, medium, low and high artifact removal.

**Fig. 3:**
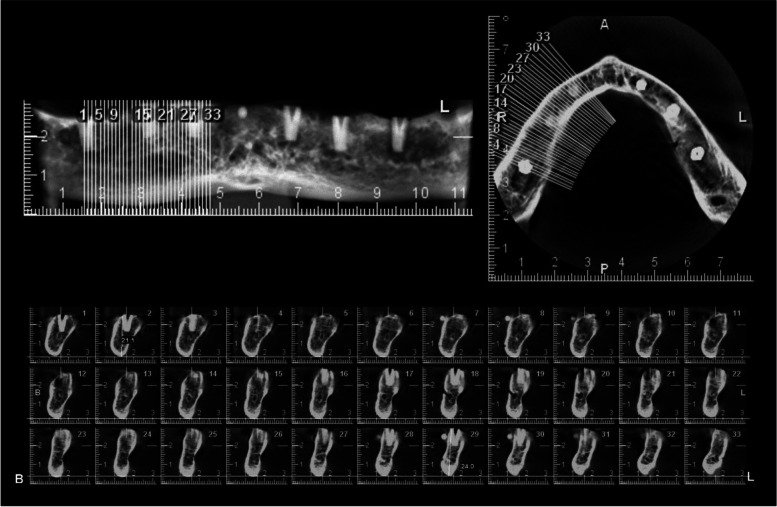
Preparation of cross sectional images

Digital caliper was used to measure the vertical height of buccal plate of each fixture as a gold standard. Cross sectional images that passes through the midline of each fixture were selected (Fig. 3). Two Oral and Maxillofacial radiologists measured the vertical buccal height blindly using Romexis 2.9.2 software under the standard lighting conditions on a 21 inches monitor (LG, Seoul, Korea). Observers could use any of the filters available in Romexis software.

Measurements were repeated at two-weeks intervals. They were requested to r SPSS version 24 (IBM, New York, USA) was used for data analysis. The difference between the measurements on software and the gold standard was calculated as the error rate. eport if the buccal crest couldn’t be observed.

Inter observer agreement was 0.83 and intra observer agreement was 0.86. Considering the high agreement, the mean error measured by two observers was considered as the overall error rate. The acceptable error rate was considered to be 0.5 mm. The generalized estimating equation model was used to analyze the accuracy of measurements. Sidak adjustment was applied for pairwise comparison of different modes. Also, the effect of voltage on each mode was evaluated. *P* < 0.05 was considered statistically significant.

## Results

In both voltages, application of ARM decreased the measurement accuracy and also it could remove the buccal crest.

Table 1 presents measurement accuracy and buccal crest visibility. If the buccal crest becomes invisible for observers due to voxel elimination by using MAR, it is considered as “missing” and if the buccal crest remains visible, it is considered as valid. For accuracy, if the difference between measurement and gold standard is equal or less than 0.5 mm it is precise and if it is more than 0.5 mm it is imprecise.

**Table 1:** Visibility of buccal crest and buccal crest height measurement error compared to the gold standard

Imprecise, > 0.5 mm	Precise, ≤ 0.5 mm	Valid	Missing	Artifact Removal	Kvp
40%(4)	60%(6)	100%(10)	0%(0)	None	54
50%(5)	40%(4)	90%(9)	10%(1)	Low	
60%(6)	10%(1)	70%(7)	30%(3)	Medium	
50%(5)	20%(2)	70%(7)	30%(3)	High	
50%(5)	50%(5)	100%(10)	0%(0)	None	58
60%(6)	40%(4)	100%(10)	0%(0)	Low	
80%(8)	20%(2)	100%(10)	0%(0)	Medium	
30%(3)	50%(5)	80%(8)	20%(2)	High	

At 54 kVp, no significant difference was found without using the ARM and low ARM (*P* = 0.568) or between high ARM and medium ARM (*P* = 1.0). However, significant differences were noted between medium/high ARMs and no/low ARM (*P* < 0.05, Table 2).

**Table 2:** Pairwise comparison of accuracy of different artifact removal modes at 54 kVp

Artifact Removal	Mean difference	*P*-Value*	95% Confidence interval for difference	
			Lower	Upper
None				
Low	0.57	0.57	-0.42	1.57
Medium	2.10	0.001	0.66	3.54
High	2.10	<0.001	0.75	3.44
Low				
None	-0.57	0.57	-1.57	0.42
Medium	1.53	0.01	0.20	2.86
High	1.53	0.003	0.37	2.68
Medium				
None	-2.10	0.001	-3.54	-0.66
Low	-1.53	0.01	-2.86	-0.20
High	-0.01	1.00	-0.34	0.32
High				
None	-2.10	< 0.001	-3.44	-0.75
Low	-1.53	0.003	-2.68	-0.37
Medium	0.01	1.00	-0.32	0.34

^*^ Sidak adjustment

At 58 kVp, no significant difference was found in accuracy of linear measurements of buccal bone height between using and non-use of ARM (*P* > 0.05, Table 3).

**Table 3:** Pairwise comparison of accuracy of different artifact removal modes at 58 kVp

Artifact Removal	Mean difference	*P*-Value*	95% Confidence interval for difference	
			Lower	Upper
None				
Low	-0.05	1.00	-0.71	0.61
Medium	0.19	0.95	-0.39	0.76
High	-0.01	1.00	-0.57	0.56
Low				
None	0.05	1.00	-0.61	0.71
Medium	0.24	0.76	-0.26	0.73
High	0.04	1.00	-0.62	0.71
Medium				
None	-0.19	0.95	-0.76	0.39
Low	-0.24	0.76	0.73	0.26
High	-0.19	0.97	-0.83	0.45
High				
None	0.01	1.00	-0.56	0.57
Low	-0.04	1.00	-0.71	0.62
Medium	0.19	0.97	-0.45	0.83

^*^ Sidak adjustment

By changing the voltage in low ARM, the accuracy of linear measurements did not change significantly (*P* = 0.351) but the difference was significant between two voltage values when medium and high ARMs were applied (*P* < 0.001), and the accuracy was higher at 58 kVp.

## Discussion

This study revealed that using MAR algorithm reduced the accuracy of linear measurement. Low ARM caused lower accuracy compared to non-use of ARM. However, this difference was not statistically significant. Medium and high ARMs decreased the accuracy of linear measurements compared to low and no ARM and the difference was statistically significant.

The structure of this algorithm is that it eliminates the voxels with gray values higher than the threshold limit (which is 8000 for low, 4000 for medium and 3500 for high ARM) [11]. Therefore, some important details may be eliminated [11]. According to Bechara and Bezarra, evaluated the diagnostic accuracy of detection of root fracture, although MAR algorithm decreased the overall image artifact, it significantly caused lower diagnostic accuracy [20, 21]. As same as present study, Parsa et al. applied titanium implants which are more popular than ceramic or zirconium ones, reported that the algorithm could not significantly correct the voxel gray value of artifacts around fixtures [2]. No significant difference was found with (low, medium, high) and without MAR in two studies by Kamburoglu et al. for detection of buccal marginal defects, periodontal defects and furcation perforations [8, 11]. According to similar studies, MAR could not significantly improve the diagnostic accuracy for detection of dehiscence and fenestration around titanium implants [10, 16]. Application of MAR is probably not suitable for fine anatomical structures that require high spatial resolution. Another study found that MAR decreased beam hardening artifact and increased image quality [22]. But higher quality did not necessary mean higher accuracy. Queiroz et al. used cylindrical utility wax phantom, reported the positive effect when artifact generating object located at the center of FOV; However, at the periphery of FOV, MAR decreased the image quality [23]. Nikbin et al. reported a similar result [19].

This study also assessed the effect of voltage on MAR algorithm and showed that increasing the voltage significantly increased the accuracy of measurements in medium and high modes. But the difference was not significant in low mode. Similar studies showed that using higher voltage decreased metal artifact [24–26]. Panjnoush et al. reported that increasing the voltage significantly decreased the metal artifact at the buccal surface of titanium rods [5]. Chindasombatjareon reported the same in both CT and CBCT [27]. Another studies found an inverse correlation between the severity of metal artifact and applied voltage [28, 29]. The majority of photons have medium level of energy and a small number of photons have maximum energy [5, 12, 30]. Thus, when hitting a high-density object such as metal, most of the photons are absorbed and a small number can pass through the object and create signals; this leads to beam hardening artifact [5, 12, 30]. By increasing the voltage, the mean energy of photons increases and consequently metal artifact would be minimized [5]. When the MAR algorithm is applied, depending on the selected mode and its threshold, voxels with higher gray value are eliminated [10, 16]. Moreover, in higher voltages, according to Bechara, the mean gray level and the gray level variation decrease while the contrast to noise ratio increases [22]. In the present in vitro study, using the voltage of 58 kVp yielded images with optimal quality and decreased the amount of artifact. With constant spatial resolution, FOV and arc rotation, increasing the voltage decreased the gray value variation and consequently by applying the high and medium ARMs, smaller details of image were lost. As a result buccal remained visible and the accuracy of linear measurement increased. However, since the low mode had a higher threshold, details was not significantly different in both voltages.

In the present study, we tried to simulate clinical condition. Hence the fixtures were located in places similar to those in the mouth. According to Misch, the canine and first molar sites are key locations for implant placement [31]. Depending on the quality of bone and location of mental foramen, first and second premolar areas may be suitable for placement of middle implants [31]; Moreover, anterior mandibular buccal crest is very thin and susceptible for resorption, which is a major complication of implant placement [31]. Thus, the central and lateral incisor, canine, premolar and first molar areas were chosen for placement of implants in our study. Moreover, other factors affecting beam hardening artifacts such as arc rotation, the reconstruction algorithm and X-ray configuration were constant in our study. Furthermore, we used dry mandible to better simulate the clinical condition comparing with homogenous phantom. Due to the fact that beam scattering may be variable when homogenous phantom was applied. However, the limitation of this study was that the phantom was positioned at the center of FOV but fixtures were located at the periphery. Another limitation was that there was no soft tissue simulation. Definitely, if soft tissue simulation was done, we could better generalize the results to the clinical situation, due to the fact that the artifacts in a cadaver or a patient may be different compared to dry mandible. But we decided to reduce the interfering factors to evaluate the pure effect of the artifact removal. The next step of the research is to evaluate the effect of other factors, including soft tissue.

## Conclusion

Although the manufacturers introduced the MAR algorithm aiming to increase the diagnostic quality of images, applying the ARM would decrease the accuracy.

As clinical condition, if high voltage is applied, application of ARM would have no significant effect on the accuracy of linear measurements.

## Data Availability

The datasets used and/or analysed during the current study are available from the corresponding author on reasonable request.

## Abbreviations

KVP: Kilovoltage peak
CBCT: Cone-beam computed tomography
CT: Computed tomography
3D: Three dimension
ARM: Artifact removal modes
MAR: Metal artifact reduction
